# Infrastructure projects and sustainable development: Discovering the stakeholders’ perception in the case of the China–Pakistan Economic Corridor

**DOI:** 10.1371/journal.pone.0237385

**Published:** 2020-08-13

**Authors:** Shahid Mahmood, Muazzam Sabir, Ghaffar Ali

**Affiliations:** 1 College of Management, Shenzhen University, Shenzhen, China; 2 College of Agriculture, University of Sargodha, Sargodha, Pakistan; Institute for Advanced Sustainability Studies, GERMANY

## Abstract

The China–Pakistan Economic Corridor (CPEC) has been an economic game changer for both China and Pakistan. We investigate stakeholder satisfaction with CPEC projects in Pakistan, particularly with respect to the affected local communities. Given the project application and adaptability, two provinces—Khyber Pakhtunkhwa and Punjab—and eight districts were selected randomly. Primary data from approximately 250 respondents as well as secondary data were collected. Among other models, a logit model was adopted to determine the role of certain factors in the perceived level of satisfaction. Results reveal that with respect to the relationship between land acquisition and dissatisfaction with a CPEC project, the level of dissatisfaction is 2.45 times higher when land is acquired by force or when compensation for the land is perceived to be inadequate by the local people. Individuals favor economic zones and development projects over road projects; the likelihood of being dissatisfied is approximately nine times greater in regions without economic zones than that in regions with such zones. Similarly, when no development project is allocated to a community, the probability of dissatisfaction of the local people is about 7.6 times greater. In addition, expectations of poor financial outcomes for CPEC projects cause significant dissatisfaction and are a source of tension among the local people. To reduce dissatisfaction, organizations and business communities must actively support the success of CPEC projects. A more equitable allocation of economic zones and development projects may help ease tensions and increase satisfaction among the local people.

## 1. Introduction

The Belt and Road Initiative (BRI), formerly known as One Belt One Road, is a global development strategy initiated in 2013 by the Chinese government to restore the paradigms of economic development along the historic Silk Road. The goal of restoring the Silk Road trade routes by connecting Asia, the Middle East, Africa, and Europe through the development of fiber optic systems, infrastructure, airports, seaports, roads, and railway networks is to provide economic benefits through trade and to empower countries that participate in this initiative with respect to their industrial, energy, and agricultural efforts [1]. The Maritime Silk Road, which focuses on developing seaports, the Silk Road Economic Belt, which connects the geographic regions through six economic corridors, and the Digital Silk Road, which encourages domestic industries to participate in the global economy, are the major components of the BRI, which will connect China and many other countries [2, 3].

An economic corridor (EC) is a network of connections between two geographic territories. The China–Pakistan Economic Corridor (CPEC) is one example of the ECs within the Silk Road Economic Belt [4, 5]. CPEC consists of free economic zones, transportation infrastructure, energy production, and infrastructure development with the goal of supporting the mutual interests of, and cooperation between, Pakistan and China. CPEC is also expected to significantly impact upon neighboring countries. To promote local connectivity, the Chinese government has focused on boosting the local economy in Pakistan, increasing investment opportunities, and developing connections with other countries [6, 7]. China aims to invest over USD$60 billion in CPEC flagship projects in Pakistan by linking the Gwadar port to Khunjerab (on the China–Pakistan border), a distance of 3,000 km, through pipelines, roads, and railway networks that will enhance trade activities throughout Pakistan [8].

The development of transportation and road infrastructures can impact upon a local community either positively or negatively. However, local business activities are also expected to benefit. CPEC development will impact upon local residents by increasing opportunities in areas such as tourism, transportation, and new businesses and will provide other economic benefits in the region [8–10]. CPEC is also expected to benefit the local community through infrastructure development, such as the building of new roads that will provide easier access to large cities, and to facilitate improvements in health, education, job opportunities, living standards, and the availability of technology, among others [8, 11]. However, few studies have examined CPEC development efforts from the perspective of the local communities regarding investment opportunities, education, health, and employment and with respect to the impact upon the community in terms of societal development [12–14]. In addition to the positive impacts of CPEC on the local community, there are some negative environmental aspects, such as water resources that are wasted owing to the construction activity, melting glaciers, noise arising from heavy machinery usage, felling of trees, etc. [6, 15].

The balance between the burdens and benefits of CPEC is highly questionable. Signs of imminent conflict are already emerging between Punjab and the less-developed provinces in Pakistan because of tensions as well as the lack of satisfaction among the local people in terms of the project activities. Local communities, primarily from Khyber Pukhtunkhwa (KPK) and Balochistan, claim a change in the CPEC route made by the government favors the Punjab and Sindh provinces with more economic zones and energy projects. Leaders of affected communities from less-developed provinces claim that they were kept out of the decision-making process, which created severe tensions among stakeholders with respect to the distribution of benefits. A significant number of people whose land has been acquired for various projects are extremely dissatisfied with CPEC because of what is seen as inadequate compensation for their land and a decline in their financial situations because of the development projects. The well-being of these people depends upon not only employment opportunities arising from road improvements but also other CPEC developments, including economic zones and projects, especially energy-related projects. Depriving the local people of what is seen as their rightful economic benefits may lead to worsened financial conditions and could be a large hurdle in the way of achieving sustainable development.

Most of the studies in this area have emphasized the importance of CPEC in various ways. Those studies also focused on pre-implementation plans, general perceptions held by the people involved, and challenges anticipated subsequent to implementing the CPEC projects. Most of the studies published have been qualitative; however, public opinion remains quantitatively low and therefore usually not sufficiently valid for measuring the impact of significant decisions. Hence, it is important to understand the level of satisfaction/dissatisfaction among local stakeholders that arises from different CPEC activities and the level of trust between local residents and CPEC administrators. This study is the first of its kind to use survey data and real-time information to investigate the level of satisfaction/dissatisfaction with respect to CPEC projects among the local communities in Pakistan. The primary focus of this study is to analyze different CPEC activities and their role in creating satisfaction or dissatisfaction among local stakeholders. These activities mainly include land acquisition and the manner in which it affects the local people’s ability to purchase alternative parcels of land of the same quality, as well as their perception about their financial situation. We also consider the impact of providing economic zones, road linkages, and other developmental projects, principally related to energy, in specific areas. The study presents policy measures and recommendations in order for the reduction of conflicts, increase in satisfaction among all stakeholders, and promotion of good governance and sustainable development. The remainder of this paper is organized as follows. The literature review is presented in Section 2. Section 3 covers the methodology used in the study. The results and discussion are presented in Section 4. Last, Section 5 offers our conclusions.

## 2. Literature review

### 2.1. Large-scale infrastructure development and associated impacts

Large-scale infrastructure projects are important to a country’s economic progress; however, such projects impose a non-trivial cost on a specific portion of the population and have major socioeconomic impacts upon the local people. Land acquisition is required for almost all large infrastructure projects, and most of the time, it is done involuntarily [16], with low compensation on offer to property owners, and with the violation of their property rights [17]. Regulations in many developing countries such as India, Pakistan, and Bangladesh do not allow individuals to challenge such land acquisitions except for the amount of compensation [18]. Displacement and resettlement give rise to several socioeconomic problems [19]. The loss of employment opportunities due to displacement is another significant drawback of such projects, often leading to social unrest and disorder [20]. The compensation people receive for having their property seized by the government is usually invested in a business that subsequently fails because of the lack of skills [21]; hence, the money is lost. Onsite jobs related to the development projects are temporary and insecure. Local people prefer public sector jobs because of their permanent nature. Otherwise, they tend to favor a longstanding or permanent facility or project that would provide new jobs to locals, thereby allowing them to secure their livelihood [22]. Public sector jobs in developing countries are typically subject to mismanagement and usually awarded by way of favoritism [23]. Projects such as those associated with CPEC are often implemented in rural and tribal areas where the local people are illiterate and lack the skills required by the projects. Raising the skills of the locals so that they can meet the demands of the onsite jobs is a necessary step if development efforts are to benefit the community [24]. This places responsibility on public authorities regarding not only governance but also the arranging and managing of training programs for affected people in order to train them to work on the projects [25]. All of these factors influence the perceptions and attitudes of locals about a project and hold the key to whether they ultimately accept or reject the project.

### 2.2. The CPEC economic game changer

**Fig 1:**
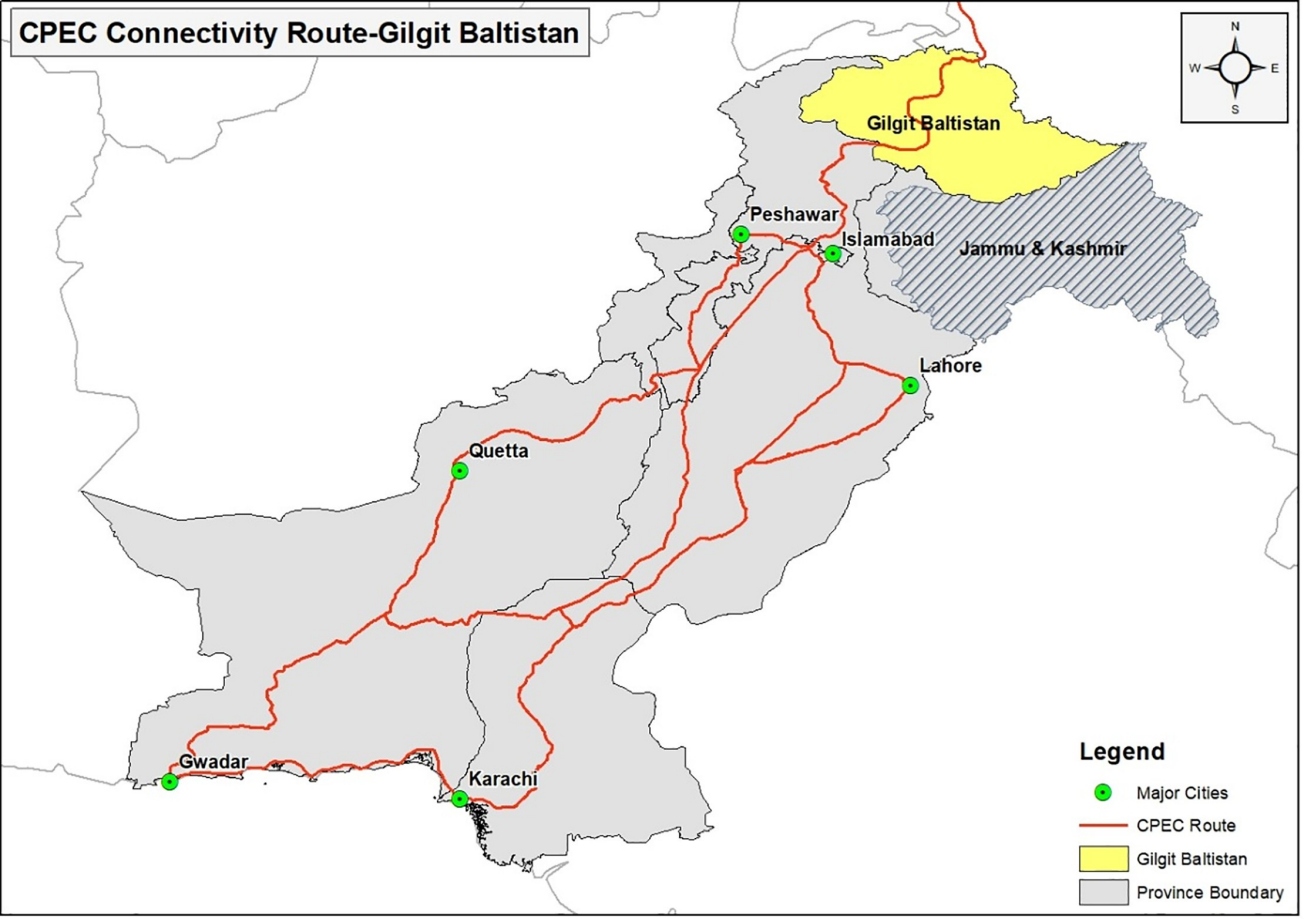
CPEC map (Silk Road Route in Pakistan from China).

The CPEC project is intended to enhance economic stability and provide other benefits to both Pakistan and China. However, building a road from the Gwadar port to Khunjerab is more beneficial for China because it is safe and is the cheapest and fastest route. It allows China to save on cost, time, and distance (a reduction from 12,500 km to 2,700 km). In addition, developing the new CPEC route provides China with an alternative to the Malacca seaport that poses a threat to Chinese businesses because of frequent incidents of piracy and armed robbery in the Strait of Malacca [26, 27]. A map of CPEC is provided in Fig 1. For Pakistan, CPEC is a game changer that is laden with many economic benefits. It will increase business, reduce poverty, generate employment opportunities, help control the country’s energy crisis, establish connectivity between rural and urban areas, attract international investors and promote industrial development, and improve health and education in the local communities [28]. The CPEC runs between Gwadar and Kashgar, passing through all of Pakistan’s provinces. Presently, CPEC projects are under development in Pakistan, including energy-related projects, Gwadar port development, road infrastructure development, special economic zone creation, mass transit development efforts in Lahore, and fiber optics. Other projects, including railways, industrial economic zone development, and an international airport in Gwadar will be implemented in the near future [4, 29]. Although CPEC has made contributions to all of the provinces of Pakistan, the allocation of benefits among the provinces is a serious concern. It is feared that as currently planned, CPEC activities will increase the disparities in the levels of development among the country’s various provinces. The lack of transparency with respect to CPEC’s policies and anticipated benefits has long been a source of conflict between Pakistan’s federal government and the provinces [30]. All of the provinces with the exception of Punjab emphasized the importance of addressing their concerns and reservations [31]. Most times, the federal government has been inclined to favor certain provinces and ignore the rights and concerns of others [32].

It is important that policymakers, government officials, and business leaders recognize the perceptions of local residents regarding CPEC development projects and the changes that need to be adopted to improve the locals’ attitudes toward CPEC.

### 2.3. CPEC development projects

The goal of CPEC from Pakistan’s perspective is to promote education, improve health and employment for its people, and help address energy issues. The investment opportunities provided through the CPEC would bring new business to local communities [33]. This would generate new employment opportunities, thereby decreasing unemployment and raising revenues for host communities [34]. With respect to tourism development, the level of satisfaction in the local community is an important factor that influences community behavior. Moreover, improving the country’s transportation infrastructure, including its system of roads, through the CPEC project would effect a positive change in the people’s standard of living, making the region more attractive, improving accessibility and quality of life, and increasing the status of local residents [14, 35]. There is a need to explore perceptions within the local community by taking initiatives, such as providing the basic necessities that would improve the quality of life. By assessing the locals’ perspectives, positive changes that would increase the social, personal, and economic well-being of the local residents could be implemented [12, 36]. CPEC would impact the welfare and lives of households by improving the community’s competitiveness and growth, e.g., by encouraging the growth of small- and medium-sized enterprises (SMEs) and import/export activities, health services, and education within the industrial zones [37, 38].

### 2.4. The CPEC and investment

CPEC development projects have the potential to create entrepreneurial ventures and investment opportunities for local residents, such as kiosks, workshops, real estate, and SMEs that can start with a low level of investment. New trade links, gas pipelines, and oil and cable projects would benefit local communities through economic and technical cooperation and the construction of industrial zones, roads, and railway networks [39]. Chinese companies and investors are eager to invest in Pakistan, which would facilitate local businesses and industries. Because of limited financial resources and entrepreneurial experience, as well as undersized market demand, it is difficult for residents of Pakistan’s rural areas to start a business; the CPEC project would enhance these opportunities in rural communities. In various ways, CPEC would reduce poverty in both rural and urban areas and could significantly impact upon economic growth. Furthermore, as local communities stabilize and improve their status, overall social conditions and quality of life would improve [40, 41].

### 2.5. Perception and level of satisfaction

Attitudes, perceptions, and the level of satisfaction among local residents have been found to support CPEC development projects. Building roads, undertaking other infrastructure improvements, and developing tourism have the most significant socioeconomic impact upon local residents [13, 42]. Researchers have suggested that the positive attitude and support of the host community are essential for the success of a project’s development. However, if road construction and other transportation-related developments lead to a loss of employment opportunities, lower living standards, human rights violations, and pollution, there could be unrest in society, which may lead to protests and conflicts [42–47]. Therefore, it is necessary to understand what the attitudes are within the local community regarding these development projects, whether locals are likely to support the CPEC projects, and how the projects are expected to influence their behaviors, attitudes, lifestyles, emotions, and well-being environmentally, culturally, socially, and economically. Agencies and public authorities responsible for implementing the projects need to take precautions to address conflicts before and after project implementation and resolve them to support effective project implementation and for the betterment of the communities involved. Thus, it is necessary to study and understand the satisfaction levels, perceptions, and conflicts among the communities involved in CPEC.

On the basis of the above literature review and discussion, we develop the following hypotheses:

**H**_**1**_: There is a relationship among categorical variables.
**H**_**o**_: There is no relationship among categorical variables.

## 3. Research methodology

### 3.1. Data collection and areas studied

To fulfill the objectives of this study, we use primary data obtained at the household level via a multistage sampling technique. We collect these data from two provinces in Pakistan, Khyber Pakhtunkhwa and Punjab. These two provinces were divided into clusters according to the districts linked to CPEC. Eight districts were randomly selected from the two provinces, namely, Faisalabad, Toba Tek Singh, Jhang, Bahawalpur, Abbottabad, Haripur, Mansehra, and Nowshera. About 266 households whose land had been occupied for any of the CPEC activities were randomly selected after several visits at the local tehsil (Tehsil is a local term used to describe “sub-district” in Pakistan) and village levels. The sample of 266 is a significant portion of the total population of approximately 1500 households that were affected across the eight districts, based on a 95% confidence interval (CI), a 5% margin of error, and a 30% response distribution as suggested by Hamburg (1935) [48]. Of the 266 questionnaires, 53 were discarded as not valid; the remaining 213 were finalized and used in the study. Responses from those households were elicited through a well-structured questionnaire. Data were collected on variables related to geographical changes and infrastructure developments resulting from the CPEC project in the selected areas. Verbal consent was taken prior to filling the form, and the data was analyzed anonymously.

Secondary sources of information were also used to supplement the results qualitatively and to help us interpret the results more effectively. This information included the literature published by government organizations about CPEC and information obtained through national and regional daily newspapers. Although these sources of information may not be typical in this type of research, they have been used in a number of previous studies [49–52]. Because this is a non-experimental, voluntary survey, no ethical approval was required.

### 3.2. Data analysis

Information gathered from the 213 valid questionnaires was used both qualitatively and quantitatively. Variables related to infrastructure development, land acquisition, and households’ land use changes were considered for quantitative analysis. Further description of the variables is presented in S1 Appendix.

We performed a Chi-square test to describe the satisfaction/dissatisfaction of respondents regarding factors related to land use changes, infrastructure development, and the financial condition of the local people. This is a common test of the relationship between categorical variables. The null hypothesis of this test states that there is no relationship between categorical variables.

$$\chi^{2}=\Sigma {\left(O-E\right)}^{2}/E,$$

where

O = observed value,

E = expected value.

A logit regression model was also used to investigate the respondents’ level of satisfaction and to further analyze the data. By using this model, we can predict the likelihood of the occurrence of an event on the basis of independent variables. It estimates the relationship between one or more explanatory variables and an output binary variable:

$$Ln\;\left(P\left(Y\right)\right)/\left(1–P\left(Y\right)\right)=\beta_{0}+\beta_{1}X_{1}+\beta_{2}X_{2}+\beta_{3}X_{3}+\beta_{4}X_{4}+\beta_{5}X_{5}+\beta_{6}X_{6}$$

where,

Y = satisfied/dissatisfied (a binary variable, where 0 = Dissatisfied and 1 = Satisfied with the overall CPEC project)

(P(Y))/(1 − P(Y)) = probability of the occurrence of the dependent (response) variable

X_1_ = land acquisition willingness. A binary variable indicating satisfaction or dissatisfaction with compensation for land (where 0 = Dissatisfied, 1 = Satisfied)

X_2_ = respondent was able to buy the same quality of land after their land was acquired for the CPEC project (where Yes = 1, No = 0)

X_3_ = a CPEC road linkage to the region/area (where Exists = 1, Does not Exist = 0)

X_4_ = a special economic zone in the region/area (where Exists = 1, Does not Exist = 0)

X_5_ = a development project (other than road and economic zone) in the specific region/area (where Exists = 1, Does not Exist = 0)

X_6_ = household perception of a change in financial situation due to the CPEC (where Improved = 1, Worsened = 0)

## 4. Results and discussion

This section presents the results obtained through analysis and discusses the findings. The results are divided into a descriptive section and other sections that address the logit model.

### 4.1. Descriptive analysis

Table 1 shows that of the 213 respondents whose land had been acquired for use by the CPEC project, 159 were not satisfied with public authorities, while 54 were satisfied. There were various reasons for which land acquisitions occurred, such as road construction, allotment for an economic stimulus zone, or an energy project for a specific area. It must be noted that appropriating land from its owners does not involve forceful eviction, with the government purchasing the land for the CPEC project. However, that purchase is not always welcome as some owners do not want to sell their land but are left with no option. Likewise, the amount of compensation paid for the land when compared with the market value could be considered to be a case of “land occupation/forced acquisition.” The governance and management of large projects usually fails to win the approval of all stakeholders [50], creating tension and conflicts [53].

**Table 1:** Frequency distribution of conflict categories.

Level of Satisfaction				
Satisfaction/ Dissatisfaction	Frequency	Percent	Cumulative Frequency	Cumulative Percent
0	159	74.65	159	74.65
1	54	25.35	213	100.00

### 4.2. Chi-square test results

Table 2 presents the results of chi-square tests between the dependent variable and the variables representing owner willingness or reluctance to sell the land for the CPEC project (X_1_) and the ability of respondents to purchase the same quality of land with the amount of compensation received (X_2_). They are statistically significant at the 5% level, which allows us to reject the null hypothesis that these results occurred by chance. Between the dependent variable and X_1_, Table 2 shows that 123 respondents were dissatisfied with public authorities overall and did not sell their land willingly. Only 29 respondents were satisfied overall but did not sell their land willingly. One possible explanation for this outcome could be that these respondents were offered a better job opportunity or the creation of an economic stimulus zone or energy project in their area that could offer better opportunities. Thirty-six respondents are dissatisfied overall with the CPEC project but nonetheless sold their land willingly. Finally, 25 of the respondents were satisfied with the CPEC and sold their land willingly.

**Table 2:** Frequency of overall satisfaction/dissatisfaction (Y) and conflicts regarding land acquisition (X_1_) and ability to purchase comparable land (X_2_).

		X_1_ by Y			X_2_ by Y	
Y		X_1_			X_2_	
**Frequency**		**0**	**1**		**0**	**1**
**0**		123	29		141	33
**1**		36	25		18	21
**Statistics**	**df**	**Value**	**Probability**	**df**	**Value**	**Probability**
**Chi-Square**	1	11.0366	0.0009	1	20.4820	< .0001
**Likelihood Ratio Chi-Square**	1	10.4600	0.0012	1	18.3227	< .0001

The results for the dependent variable X_2_ show that 141 respondents were dissatisfied with the public authorities overall and had not been able to buy land of quality equivalent to that of the land they were required to sell to the CPEC. Thirty-three respondents were satisfied overall but could not purchase land of the same quality. Eighteen respondents were satisfied overall even though they had been unable to buy land of the same quality. Finally, 21 respondents were generally satisfied with the authorities regarding the CPEC and had been able to buy land of the same quality. Overall, the results are mixed, as some level of satisfaction existed, while at the same time, many were dissatisfied. The results also reflect the fact that the quality of land differs in those provinces and districts and that this is a concern for old tenants of the land when they need to buy a new piece of property. Land compensation is a major issue in these types of projects, and inadequate or delayed compensation leads to dissatisfaction among stakeholders, creating tension and conflicts [54]. These projects are often brought to remote and tribal areas where locals do not have legal rights to land. This situation reduces the compensation paid to the locals for their land [55]. In most developing countries, including India, Pakistan, and Bangladesh, only the amount of compensation can be challenged but not the land acquisition itself [56]. Buying land of the same quality that provides the same domestic and cultural setup becomes almost impossible in such situations [56].

Table 3 presents the chi-square test results between the dependent variable and independent variables X_3_ and X_4_. The variable X_4_ is statistically significant at the 5% level, allowing us to reject the null hypothesis that the results occurred by chance. However, X_3_ lacks significance and Table 3 shows that for 33 respondents, their region/area was not linked to road projects under the CPEC and that they were dissatisfied with the overall CPEC project. Nineteen respondents were satisfied even though there was no road linking the CPEC to their area. Of the 152 respondents who were dissatisfied with the CPEC, 119 had a road linking the CPEC to their area. Finally, 42 of the respondents were satisfied with the overall CPEC project and also had a road link to their area.

**Table 3:** Frequency of overall satisfaction/dissatisfaction (Y) and existence of road links (X_3_) and economic zones (X_4_).

		X_3_ by Y			X_4_ by Y	
Y		X_3_			X_4_	
**Frequency**		**0**	**1**		**0**	**1**
**0**		33	19		103	35
**1**		119	42		49	26
**Statistics**	**df**	**Value**	**Probability**	**df**	**Value**	**Probability**
**Chi-Square**	1	2.1008	0.1472	1	9.7583	0.0015
**Likelihood Ratio Chi-Square**	1	2.0364	0.1536	1	9.3269	0.0019

The results of the analysis for the dependent variable and X_4_ show that 103 respondents who had no special economic zone in their area were dissatisfied with the CPEC project. Thirty-five respondents were satisfied even without having a special economic zone in their area. Forty-nine respondents were dissatisfied with CPEC overall but had an economic zone in their area. Finally, 26 respondents were both satisfied with the CPEC project and had an economic zone in their area. Although several people suffered a loss of employment due to the large infrastructure and development projects, these projects bring many employment opportunities to an area. Still, jobs associated with infrastructure construction are temporary and insecure by nature. Local people affected by these projects prefer secure and permanent jobs [55]. The construction of special economic zones and energy projects could provide long-term employment opportunities that are more secure than jobs arising from road construction projects.

Table 4 presents the chi-square test results between the dependent variable and independent variables X_5_ and X_6_, which represent the existence of development projects in the area and respondents’ satisfaction with changes in their financial situation, respectively. Both are statistically significant at the 5% level, thus rejecting the null hypothesis that the results occurred by chance. In considering the relationship between the dependent variable and X_5_, the results show general dissatisfaction with the CPEC administration on the part of 116 respondents who had no development projects other than the CPEC in their area. In contrast, 27 respondents were generally satisfied even though they had no other development project in their area. Forty-three respondents were dissatisfied overall even though they did have a development project in their area. Finally, 27 respondents were satisfied overall with the CPEC project and also had a development project in their area.

**Table 4:** Frequency of overall satisfaction/dissatisfaction (Y) and existence of development projects (X_5_) and financial condition of respondents (X_6_).

		X_5_ by Y			X_6_ by Y	
Y		X_5_			X_6_	
**Frequency**		**0**	**1**		**0**	**1**
**0**		116	27		81	22
**1**		43	27		71	39
**Statistics**	**df**	**Value**	**Probability**	**df**	**Value**	**Probability**
**Chi-Square**	1	9.6278	0.0019	1	5.1711	0.0230
**Likelihood Ratio Chi-Square**	1	9.2723	0.0023	1	5.2293	0.0222

The results for the dependent variable and X_6_ show that 81 respondents were dissatisfied with changes in their financial situation because of the CPEC and were dissatisfied overall with the public authorities. Twenty-two respondents were satisfied overall but were dissatisfied with the degree of improvement (or lack thereof) with respect to their personal financial situation. Seventy-one respondents were dissatisfied with the CPEC project overall but were satisfied with their financial situation. Finally, 39 respondents were satisfied overall and were also satisfied with their financial situation. The governance and management of large-scale projects should be done such that they improve the living standard of the local people [54]; however, such projects often have a negative impact on an individual’s standard of living, particularly in developing countries [19]. People are not always able to maintain their original profession [55], and the forced displacement [16] and inadequate compensation for land appropriated for the project degrade their financial situation [56]. Because these projects are typically brought to remote and tribal areas where a majority of locals are illiterate and unskilled, without some type of training, they cannot even maintain their original financial condition, let alone benefit from the project [50].

### 4.3. Logit regression model results

Table 5 presents the results of the binary logit regression analysis, where the level of dissatisfaction is modeled as “0.” This probability of dissatisfaction was tested against the independent variables as shown in Table 5. The relationship between land being acquired (X_1_) and the dependent variable shows that the probability of being dissatisfied was 2.45 times higher when land was appropriated for the project or when the local people were not satisfied with the amount of compensation offered to them for their land. Similarly, the probability of being dissatisfied was about seven times higher when the affected local respondent was not able to buy land of the same quality as the one that was taken by the government for the CPEC project. Regarding the link to the CPEC road, the odds of being dissatisfied were about 0.3 times lower when there was no road link to their area but their land was taken for some other CPEC-related projects or economic zones. That there was no road link meant that the area had an economic zone or some other development project providing local people with better economic opportunities, which reduced the likelihood of conflict caused by the dearth of a road link. These findings support the view that people in these regions are less interested in road projects and are more interested in economic zones and other projects. Table 6 shows the significance of the model.

**Table 5:** Analysis of maximum likelihood estimates.

Parameter		df	Estimate	Standard Error	Wald Chi-Square	Pr > ChiSq	Odds Ratio
**Intercept**		1	1.0791	0.5255	4.2174	0.0400	
**X**_**1**_	**0**	1	0.4482	0.1912	5.4984	0.0190	2.451
**X**_**2**_	**0**	1	0.9886	0.2308	18.3451	< .0001	7.223
**X**_**3**_		1	−1.2159	0.5997	4.1105	0.0426	0.296
**X**_**4**_	**0**	1	1.1058	0.3309	11.1684	0.0008	9.131
**X**_**5**_	**0**	1	1.0195	0.2634	14.9809	0.0001	7.684
**X**_**6**_		1	0.5331	0.3713	2.0613	0.1511	0.587

**Table 6:** Significance of model (testing global null hypothesis: BETA = 0).

Test	Chi-Square	df	Pr > ChiSq
**Likelihood Ratio**	48.5589	6	< .0001
**Score**	46.3669	6	< .0001
**Wald**	33.1548	6	< .0001

**Fig 2:**
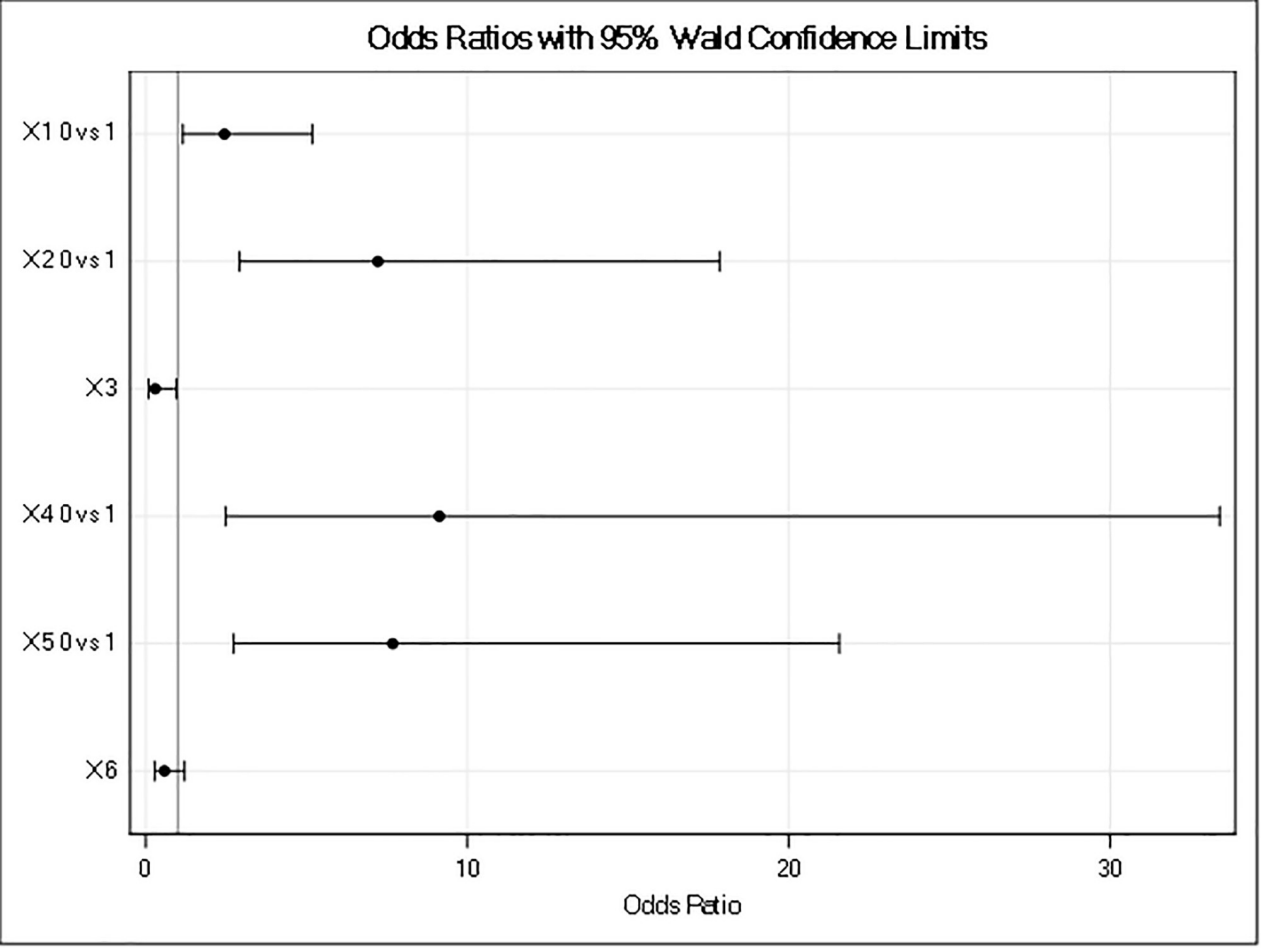
Odds ratios (independent variables).

**Fig 3:**
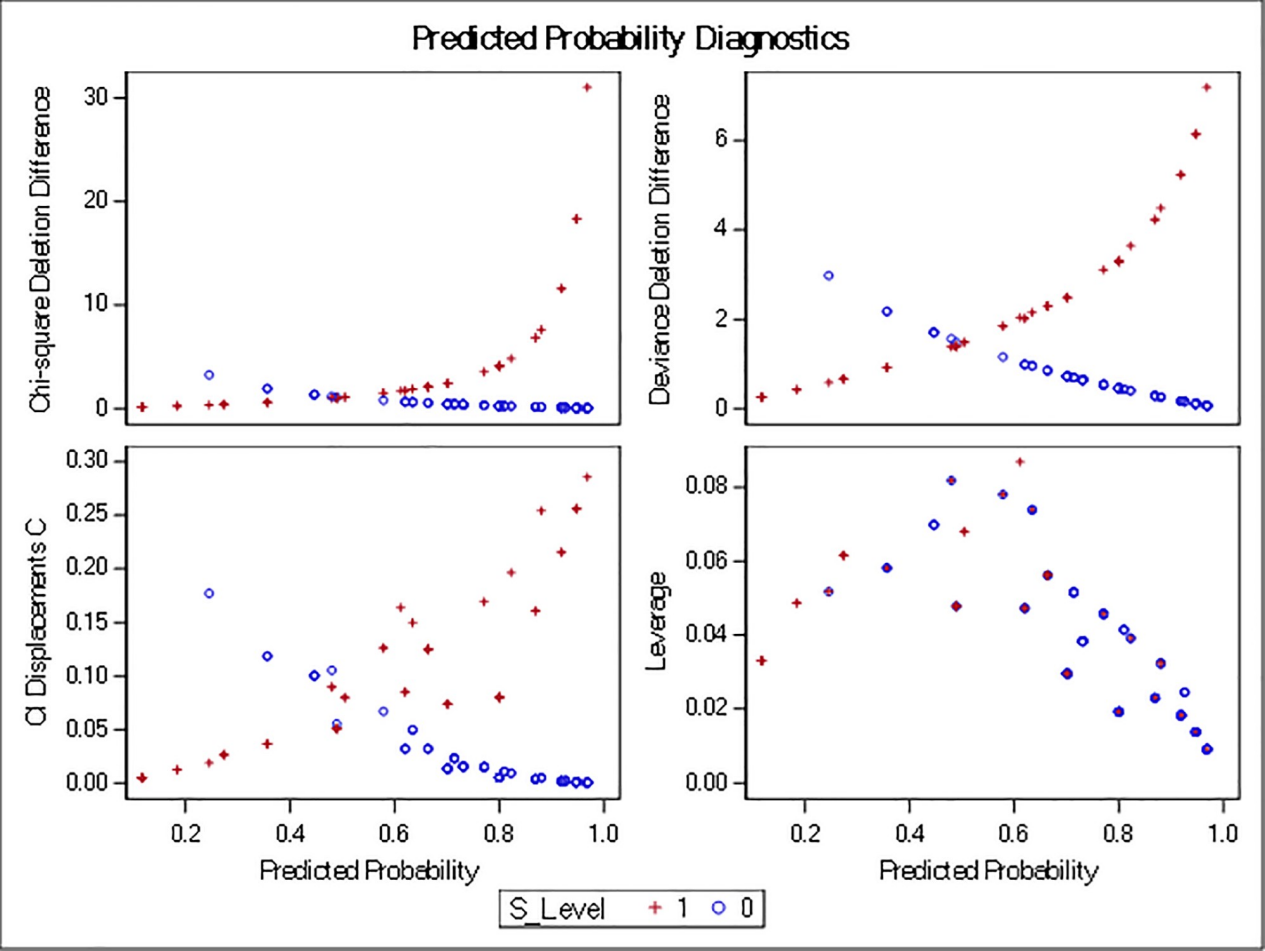
Predicted probability diagnostic of the model.

**Fig 4:**
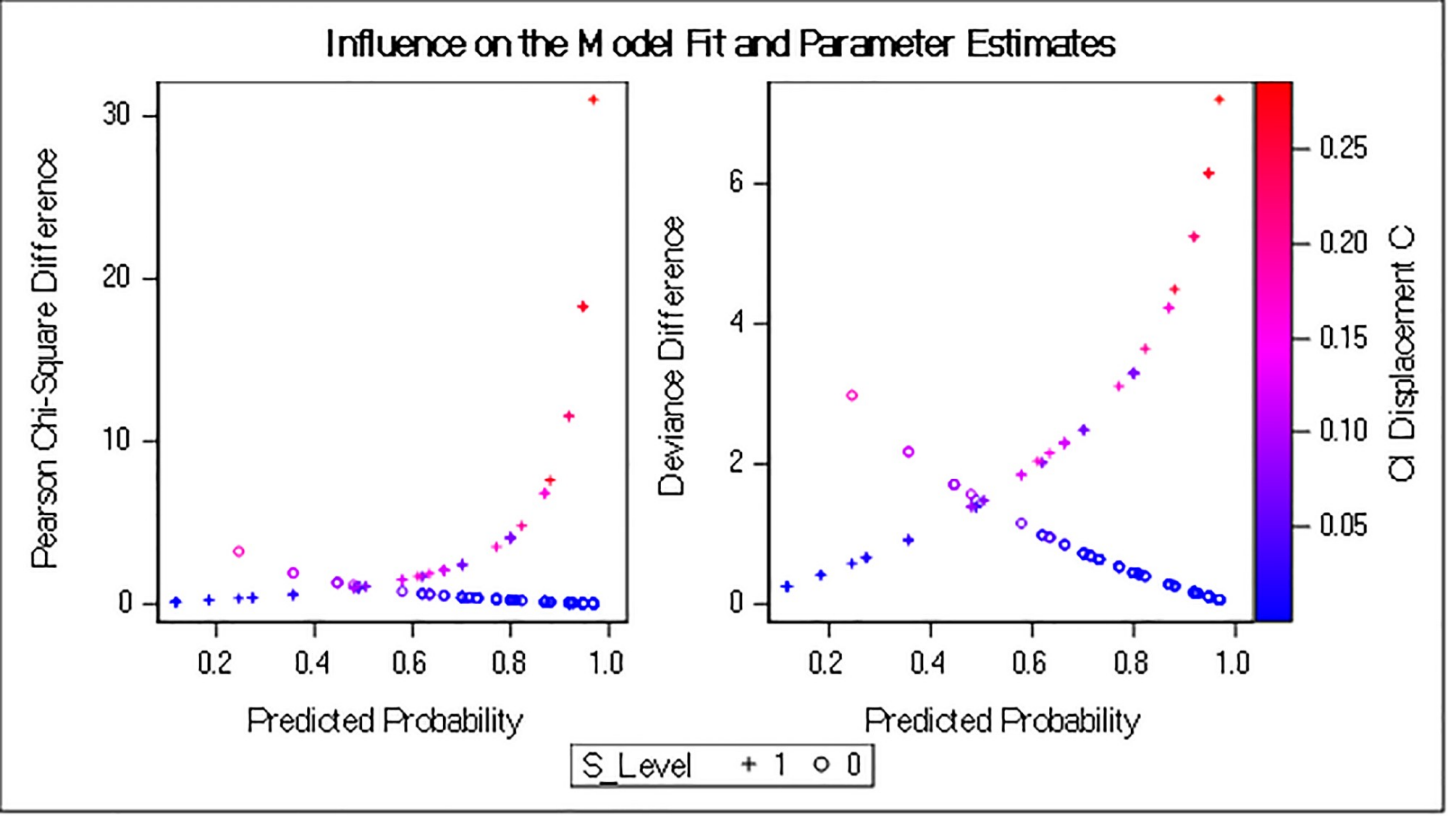
Influence on model fit and parameter estimations.

**Fig 5:**
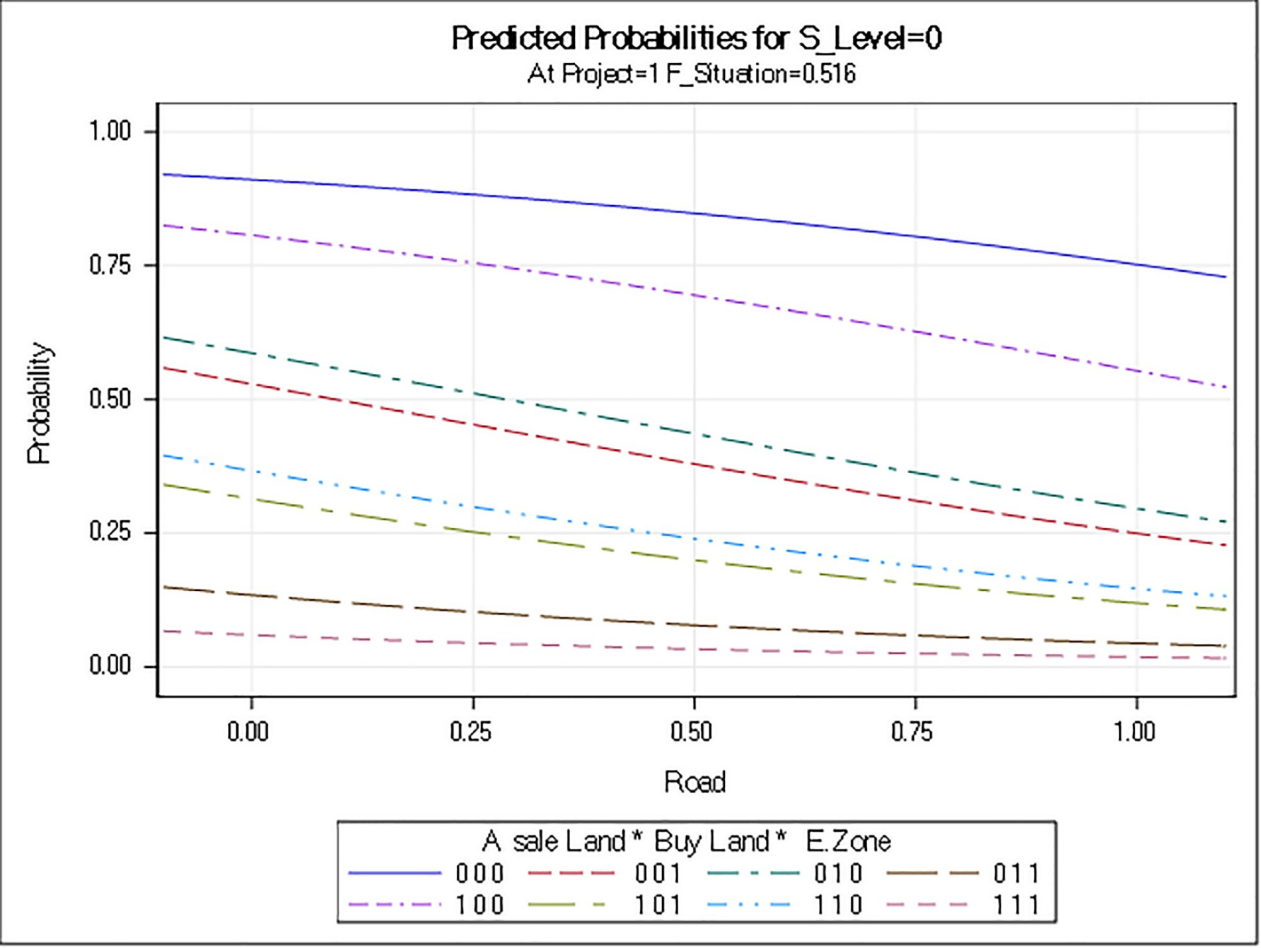
Prediction of probabilities for dissatisfaction in areas studied.

The regions with no economic zones were about nine times more likely to be dissatisfied with CPEC overall than regions that had an economic zone. Similarly, the odds of being dissatisfied were about 7.6 times higher when there was no development project allocated to the area. Furthermore, people’s expectations of a poor financial outcome due to CPEC cause them to be dissatisfied and could create conflicts. Odds ratios are plotted in Fig 2, which combines the level of dissatisfaction with different factors (independent variables) that can contribute to conflict. Figs 3–5 show the predicted probability diagnostics, the model's goodness of fit, and predicted probabilities of dissatisfaction in the areas studied and the data that were analyzed. All of these results were extracted from the logit regression model.

## 5. Conclusions

This study focused on whether people in selected regions of Pakistan were generally satisfied or dissatisfied with CPEC. We explained as to whether dissatisfaction existed with respect to different CPEC project activities and the manner in which the local people perceived them. We also tested our two hypotheses, and the null hypothesis that there is no relationship between our categorical variables was rejected in each case. It was interesting to see that some of the people who were directly or indirectly involved in the CPEC project were satisfied and some were dissatisfied. A minority of locals surveyed sold their lands to the CPEC project willingly, and land acquisition occurred smoothly regardless of whether or not they were able to acquire land of equivalent quality. There were also people who sold their land unwillingly but were still satisfied with the project. This satisfaction was highly dependent upon the availability of job opportunities through economic zones and/or energy projects, as their areas had been allocated special economic zones and energy projects that provided them with good job opportunities. However, a number of them sold their land unwillingly, were generally dissatisfied with the public authorities, and had concerns regarding the distribution of economic zones within their regions. Moreover, some of the respondents were concerned regarding the allocation of development projects and roads in their areas as they expected economic opportunities to arise with the arrival of CPEC. A majority of the people were dissatisfied with respect to improvements in their financial situation because of the opening of CPEC in Pakistan although some were still satisfied with the CPEC overall. Therefore, the situation could be said to be quite mixed throughout the areas studied. More clarity, based on and backed by further analysis, may arise after complete implementation of the CPEC project.

Nonetheless, on the basis of our results, we believe that the distribution of economic zones and energy projects, particularly their equal distribution, and the participation of all stakeholders in decision-making could play a significant role in conflict mitigation and satisfaction for all stakeholders. Our results have a number of policy recommendations, which are as follows: governments, private organizations, and business communities have a significant role in making the CPEC projects successful. Public officials should try to resolve situations that give rise to conflicts as early as possible. Distribution and allocation of economic zones should be fair and equitable. This study has certain limitations. While multiple variables and factors were considered in this study, other factors related to infrastructure, environment, energy, and the local and agricultural sectors could be considered in future studies. The results of the study and the method of analysis could be applied to many other BRI countries for, and in anticipation of, post-implementation work related to various projects. In addition, from the Chinese perspective, this type of study should be conducted to analyze their ability to improve outcomes for the BRI.

## Supporting information

S1 Appendix (DOCX)

S1 Data (XLSX)

S1 File (PDF)
