# Supplementary material for: Infrastructure projects and sustainable development: Discovering the stakeholders’ perception in the case of the China–Pakistan Economic Corridor
Source: PLoS One. 2020 Aug 13;15(8):e0237385. doi: 10.1371/journal.pone.0237385 (PMC7425984; doi:10.1371/journal.pone.0237385)
Supplement: S1 Appendix — (DOCX) [file pone.0237385.s001.docx]

S1 Appendix.

Y = satisfied/dissatisfied (a binary variable, where 0= Dissatisfied and 1 = Satisfied with the overall CPEC project).

X_1_ = land acquisition willingness. A binary variable indicating satisfaction or dissatisfaction with compensation for land (where 0 = Dissatisfied, 1 = Satisfied).

X_2_ = respondent was able to buy the same quality of land after his/her land was acquired for the CPEC project (where Yes = 1, No = 0).

X_3_ = a CPEC road linkage to the region/area (where Exists = 1, Does not Exist = 0).

X_4_ = a special economic zone in the region/area (where Exists = 1, Does not Exist = 0).

X_5_ = a development project (other than road and economic zone) in the specific region/area (where Exists = 1, Does not Exist = 0).

X_6_ = household perception of a change in financial situation due to the CPEC (where Improved = 1, Worsened = 0).
