# Supplementary material for: Infrastructure projects and sustainable development: Discovering the stakeholders’ perception in the case of the China–Pakistan Economic Corridor
Source: PLoS One. 2020 Aug 13;15(8):e0237385. doi: 10.1371/journal.pone.0237385 (PMC7425984; doi:10.1371/journal.pone.0237385)
Supplement: S1 File — (PDF) [file pone.0237385.s003.pdf]

Questionnaire

Dear respondent, we are conducting a research on **Socio-economic and Socio-environmental assessment of China-Pakistan Economic Corridor: Key stakeholders' analysis from Pakistan side**.

In order to complete this research-work, I need your voluntary participation in this survey. If you are willing to participate in this study, then I request you to respond to the statements given as under of this Short Note. Your name should not appear anywhere in this survey, and your response will be treated confidentially and will only be used for research purpose. Your valuable response will help us in the completion of this project.

1. Gender: ☐ Male ☐ Female ☐ Other

2. Marital status ☐Single ☐ Married ☐ Divorced

3. Language ☐ Urdu ☐English ☐Other please specify

4. Respondent ☐ Household head ☐ Family member

5. If family member please specify the relationship with household……………..

6. Age: Please specify (   )

7. Occupation:
   ☐ Businessman ☐ Government Employee ☐ Private Employee
   ☐ Student ☐ Unemployed ☐ Household

8. Education
   ☐ PhD or Above ☐ Masters ☐ Graduation
   ☐ Higher Secondary ☐ Matric ☐ Less than Matric

9. Income level Please specify (   )

10. Household size: Total number of household members and characteristics.

S.no	Name	Gender	Age	Relation to head/respondent	Marital status	Education	Occupation
1							
2							
3							
4							
5							
6							
7							

11. Do you do any subsistence production?

Fruit/Vegetables	Egg/Milk/Milk Products	Meat	Other

12. How much you spend monthly on food/drink? Please specify (   )

13. How much you spend monthly on Utilities (Electricity etc)? Please specify (   )

14. How much you spend monthly on Health? Please specify (   )

15. How much you spend monthly on Education? Please specify (   )

16. Other Expenditure (Please specify)? (   )

17. How much you save? (   )

18. Do you think your financial situation could improve due to CPEC development?

☐ Yes ☐ no

19. If yes then how much could be the impact (positive or negative)?

Increase	Decrease
☐ 0 -20% ☐ 21 - 40%	☐ 0 -20% ☐ 21 - 40%
☐ 41 - 60% ☐61 - 80%	☐ 41 - 60% ☐61 - 80%
☐ 81 - 100%	☐ 81 - 100%

20. Do you think the immigration of foreigners will bring any positive or negative impact on local community lifestyle?

☐ Yes ☐ No

21. If yes then how much will be the impact (positive or negative)?

Positive impact	Negative impact
☐ 0 -20% ☐ 21 - 40%	☐ 0 -20% ☐ 21 - 40%
☐ 41 - 60% ☐61 - 80%	☐ 41 - 60% ☐61 - 80%
☐ 81 - 100%	☐ 81 - 100%
☐ 0 -20% ☐ 21 - 40%	☐ 0 -20% ☐ 21 - 40%

22. Does your land have been used or occupied during the construction of the CPEC projects?

☐ Yes ☐ No

23. If yes please answer the following questions.

24. How much area of land?

☐ Less than 1 kanal ☐ [illegible] kanal to 2 kanal [illegible]☐kanal to 4 kanal

☐ 4 kanal to 6 kanal ☐[illegible] kanal to 8 kanal [illegible]☐ore than 8 kanal

25. Did you sale the land willingly?

☐ Yes ☐ No

26. What is the market value of land? Please specify (   )

27. How much you have received? Please specify (   )

28. Your expected price of this land? Please specify (   )

29. Before occupation of CPEC for which purpose you had used this land?
   ☐ Businessman ☐ Agriculture ☐ Household ☐ other

30. Can you buy same quality of land at other place with this compensation?
   ☐ Yes ☐ No
31. Are you satisfied after selling this land?
   ☐ Yes ☐ No
32. Do you have any offer to work in the project?
   ☐ Yes ☐ No
33. Under CPEC what kind of measures are going to be held in your area for rural development?
   ☐ Construction of Infrastructure ☐ Rural Electrification
   ☐ Improving agricultural sector ☐ Increasing and improved irrigation system
34. Are you satisfied with overall CPEC project?
   Yes No
35. If not, do you plan to respond to Government in any way?

Thanks for your Time.
